# Investigating the efficacy of an individualized metacognitive therapy program (MCT+) for psychosis: study protocol of a multi-center randomized controlled trial

**DOI:** 10.1186/s12888-016-0756-2

**Published:** 2016-02-27

**Authors:** Brooke C. Schneider, Martin Brüne, Francesca Bohn, Ruth Veckenstedt, Katharina Kolbeck, Eva Krieger, Anna Becker, Kim Alisha Drommelschmidt, Susanne Englisch, Sarah Eisenacher, Sie-In Lee-Grimm, Matthias Nagel, Mathias Zink, Steffen Moritz

**Affiliations:** Department of Psychiatry and Psychotherapy, University Medical Center Hamburg-Eppendorf, Hamburg, Germany; LWL University Hospital Bochum, Department of Psychiatry, Psychotherapy and Preventative Medicine, Division of Cognitive Neuropsychiatry and Psychiatric Preventative Medicine, Ruhr-University Bochum, Bochum, Germany; Department of Psychiatry and Psychotherapy, Asklepios North-Wandsbek, Hamburg, Germany; Department of Psychiatry and Psychotherapy, Central Institute of Mental Health, Medical Faculty Mannheim, Heidelberg University, Mannheim, Germany; Department of Psychiatry and Psychotherapy, University of Lübeck, Lübeck, Germany

**Keywords:** Schizophrenia, Metacognitive training, Psychosis, Cognitive biases, Delusions

## Abstract

**Background:**

Psychological interventions are increasingly recommended as adjunctive treatments for psychosis, but their implementation in clinical practice is still insufficient. The individualized metacognitive therapy program (MCT+; www.uke.de/mct_plus) represents a low-threshold psychotherapeutic approach that synthesizes group metacognitive training (MCT) and cognitive behavioral therapy for psychosis, and addresses specific cognitive biases that are involved in the onset and maintenance of psychosis. It aims to “plant the seed of doubt” regarding rigid delusional convictions and to encourage patients to critically reflect, extend and change their approach to problem solving. Its second edition also puts more emphasis on affective symptoms. A recent meta-analysis of metacognitive interventions (MCT, MCT+) indicate small to moderate effects on positive symptoms and delusions, as well as high rates of acceptance. Nonetheless, no long-term studies of MCT+ involving large samples have been conducted.

**Methods:**

The goal of the present multi-center, observer-blind, parallel-group, randomized controlled trial is to compare the efficacy of MCT+ against an active control (cognitive remediation; MyBrainTraining^©^) in 328 patients with psychosis at three time points (baseline, immediately after intervention [6 weeks] and 6 months later). The primary outcome is change in psychosis symptoms over the 6-month follow-up period as assessed by the delusion subscale of the Psychotic Symptom Rating Scale. Secondary outcomes include jumping to conclusions, other positive symptoms of schizophrenia, depressive symptoms, self-esteem, quality of life, and cognitive insight. The study also seeks to elucidate mediating factors that promote versus impede symptom improvement across time.

**Discussion:**

This is the first multi-center randomized controlled trial to test the efficacy of individualized MCT+ in a large sample of patients with psychosis. The rationale for the trial, the design, and the strengths and limitations of the study are discussed.

**Trial Registration:**

The trial is registered through the German Clinical Trials Register (www.drks.de) as DRKS00008001. Registered 6 May 2015.

## Background

Schizophrenia remains a stigmatizing and chronic disorder that affects approximately 1 % of the population worldwide and is among the world’s top ten causes of chronic disability [1]. Due to the poor quality of life among many of those affected [2] and the large healthcare costs incurred through repeated hospitalizations [3], improved treatment for schizophrenia is needed. Although antipsychotic medications remain the treatment of choice for schizophrenia, up to 60 % of patients do not respond to antipsychotic medications [3] and rates of non-adherence are as high as 60 % within the first year of treatment [4]. Furthermore, among those who are compliant with medications, a minority (approximately 40 %) have a good symptomatic outcome and recovery rates are only around 20 %. Moderate effect sizes have been reported for improvement in positive symptoms relative to placebo; however, non-pharmacological approaches are also necessary to treat cognitive symptoms, such as cognitive biases [5–7].

Given the limitations to treatment with antipsychotic medications alone, there has been increasing interest in developing alternative interventions for psychosis, and particularly adjunctive psychotherapies have been integrated as a core element of international treatment guidelines [8]. Cognitive behavioral therapy for psychosis (CBTp) [9–12] and cognitive remediation (CR) [13] represent two such psychotherapeutic approaches, which have yielded small to medium effects beyond antipsychotic medications. While CBTp aims to identify pathological symptoms and change dysfunctional cognitive processes, CR focuses on improvement of deficits in basic neuropsychological functioning (i.e., memory, executive functioning attention) through computerized or paper-and-pencil exercises. Despite the promising support for these interventions and evidence that patients with psychosis are responsive to psychotherapy, a majority of patients never receive empirically-based treatments (EBTs) ([14, 15], Moritz S, Berna F, Jaeger S, Westermann S, Nagel M. The customer is always right? Subjective target symptoms and treatment preferences in patients with psychosis, submitted.) due to a number of patient (e.g., stigma, shame) and organizational barriers. Among those patients who do seek treatment, many must wait several months before they can begin therapy due to limitations in insurance reimbursement or therapist availability. Additionally, while therapists and family members identify positive symptoms as a main treatment goal, patients tend to prioritize rather quality of life and affective symptoms [(Moritz S, Berna F, Jaeger S, Westermann S, Nagel M. The customer is always right? Subjective target symptoms and treatment preferences in patients with psychosis, submitted) 16, 17]. As such, lack of agreement on treatment goals between therapists and patients may also contribute to poorer therapy adherence and satisfaction. At the organizational level, therapists cite a lack of funding to gain necessary training, as well as negative staff attitudes toward EBTs. Many therapists feel that they do not have sufficient time to appropriately implement EBTs, despite adequate training [15]. As such, there is need for effective low-intensity interventions, which can increase treatment accessibility for patients with psychosis [18].

The current study is designed to test the effectiveness of an innovative treatment for psychosis, the individualized metacognitive therapy program (MCT+), compared to an active control group, which completes cognitive remediation. The group version of the treatment program, metacognitive training (MCT) was developed by Moritz and colleagues in 2002/2003 and adopts a metacognitive perspective (i.e., “thinking about one’s thinking”). The MCT approach is based on significant evidence from many research groups [19–25], including our own [26–29], that cognitive biases underlie the formation and maintenance of schizophrenia. While patients are often unaware of these cognitive biases (such as overconfidence, incorrigibility and hasty decision-making), they are not limited only to delusional beliefs, but can also be observed in daily delusion-neutral situations. As discussed in recent publications [30, 31], MCT aims to “plant the seed of doubt” regarding delusions through corrective (“aha!”) experiences in an entertaining, playful and collaborative manner. In this way, MCT aims to raise patients’ awareness regarding the negative impact of cognitive biases, which contribute to the pathogenesis and perpetuation of positive symptoms.

The intervention borrows from CBTp, CR and psychoeducational approaches by focusing on symptoms, presenting multiple cognitive exercises and providing general information on the disorder. However, differing from CBTp, MCT adopts a “backdoor” approach in which general cognitive processes are first addressed before examining how these biases may lead to problems in everyday life and psychotic symptoms, respectively. Additionally, rather than focusing on accuracy and traditional neuropsychological faculties (e.g., memory, attention) as in CR, MCT particularly attempts to attenuate participants’ overconfidence in errors (e.g., by asking patients to consider their level of certainty before making a final decision) and delay decision-making for momentous judgments. Therefore, the goal of MCT is not to dissuade patients from their delusional thoughts, but to promote doubt regarding the likelihood that the delusional thought content accurately reflects reality.

The individualized version of MCT, termed MCT+, was recently developed to allow for more detailed targeting of patient-specific problems, which cannot be addressed in group therapy [32, 33]. Importantly, overcoming the above-mentioned barriers to psychotherapy, MCT+ is short (up to 12 sessions), but has an evidence-based mechanism of action, and although it is highly structured and fully manualized, the therapist can select the therapy units that fit best to the patient’s current difficulties and cognitive biases. Based on patient feedback and research findings that patients wish to prioritize treatment of affective symptoms, in the newest edition, more focus has been placed on affective symptoms. Like the group variant, in MCT+ therapists first teach participants about cognitive biases and dysfunctional emotional regulation before individual problems are addressed. To increase the dissemination and use of MCT and MCT+; materials are available for free in many world languages. The group MCT is available in 33 language versions and MCT+ is currently available in 10 versions.

As summarized in a comprehensive review [30], the feasibility, safety and efficacy of group MCT has been demonstrated in several study trials. The intervention is well-accepted by patients, who indicate that they find the training to be fun and would recommend MCT to others [30, 34, 35]. A recent meta-analysis [35] revealed that MCT yields significant small to moderate effects on positive symptoms (*g* = .34) and delusions (*g* = .41) in comparison to control conditions and that effects are higher for the three completed trials of individualized MCT (positive symptoms: *g* = −0.53; delusions: *g* = −0.26). However, it is important to point out that two of the studies included in these analyses utilized the materials for group MCT in an individualized setting [36–38]. Therefore, there has been only one trial of MCT+ [34], which yielded small to moderate significant improvements (η_partial_^*2*^ = .10) in delusions and positive symptoms compared to an active control group. Despite the overall positive effects, notably, one large trial by van Oosterhout and colleagues (2014) found no superiority for group MCT compared to a treatment-as-usual (TAU) control group for symptoms. Although the reasons for this “outlier” remain unclear, the study included patients with moderate to severe delusions and used an older version of group MCT, which did not highlight the role of (over) confidence. While the training is aimed at improving positive symptoms, clinical experience indicates that patients with acute symptoms often have difficulty engaging in the group setting and have limited ability to acknowledge cognitive biases. Therefore, it is now recommended that trainers screen out such patients for the group intervention. Such patients may be more amenable to MCT+ as the individualized setting is more flexible and allows more time for addressing such acute symptoms. Additionally, the earlier version of MCT did not include as much focus on attenuating confidence in the face of incomplete or ambiguous evidence, which is now a core component of the training. Despite the possible explanations for these null findings, it must also be considered that MCT is not helpful for all patients [39].

While some studies have demonstrated significant improvements in cognitive biases (especially jumping to conclusions or data gathering) thought to be implicated in delusional ideation [40–45], others have not been able to find significant differences [36, 46, 47] in comparison to active control and wait-list groups. Recent studies revealed significant improvements in cognitive insight for MCT compared to an active control group [48] and a wait-list control group [46]; however, no significant differences were reported in the van Oosterhout et al. trial. One trial found a significant group difference for improvement in social functioning [49]; however, in this study, MCT was combined with social cognition training.

Despite these promising findings, the long-term effects of MCT and particularly MCT+ remain largely unknown. In one recent MCT trial, improvement on the PSYRATS was sustained for up to three years, and “sleeper effects” for self-esteem and global quality of life emerged only at the three year follow-up [47]. Nonetheless, there has only been one trial of MCT+, which was small (*n* = 48) and did not involve follow-up assessment. Moreover, MCT+ has been updated since this study and now includes more focus on affective symptoms and stigma. A large trial is also needed to elucidate for which symptom domain MCT+ is the most helpful and symptoms or demographic constellations (e.g., age, gender, education) that may moderate outcome.

### Aims of the trial

The primary aim of this study is to investigate the effectiveness of MCT+ versus computerized cognitive remediation (CR; MyBrainTraining^©^) in individuals with psychosis.

The primary research question is:

1. Does MCT+ result in a greater reduction in rater-observed symptoms of delusions, as measured by the Psychotic Symptom Rating Scales (PSYRATS delusions subscale), compared to computerized CR at the end of treatment (6 weeks)?

Secondary research questions are:

2) Does MCT+ result in a greater reduction in other positive, negative and affective psychopathology than computerized CR as measured by the Positive and Negative Symptoms Scales (PANSS)?
3) Does MCT+ result in a greater reduction in jumping to conclusions than computerized CR? Is change in jumping to conclusions associated with improvement in positive symptoms?
4) Does MCT+ result in a greater reduction in self-reported symptoms of depression and/or a greater increase in quality of life, self-esteem, and cognitive insight than computerized CR?
5) Does computerized CR result in a greater improvement in neuropsychological functioning than MCT+?
6) Are baseline characteristics or therapy motivation predictive of treatment response to MCT+?

Although small, yet significant, decreases in psychosis symptoms have been reported in some studies of CR interventions [13], based on previous work, we hypothesize that MCT+ will lead to a greater decrease in delusions as compared to patients in the computerized CR group at post and follow-up test sessions (Q1). Cognitive remediation is deemed an appropriate comparison group for the present study since its feasibility has been asserted, and because it offers some amelioration of neuropsychological symptoms, but does not address cognitive biases at the core of MCT+. We further hypothesize that, compared to the control group, we will find a greater reduction in the PANSS positive syndrome [50] at the same time points for MCT+ participants (Q2). Given previous mixed results regarding improvement in jumping to conclusions [30, 51], we hypothesize that data gathering will improve in both groups, but that only improvement in the MCT+ group will be significantly associated with improvement in delusions (Q3). Because the newest version of MCT+ places more focus on affective symptoms and significant (sleeper) effects have already been detected for quality of life and self-esteem in group MCT (which did not include these new therapy units), we anticipate that participants who complete MCT+ will demonstrate improvements in depressive symptoms, quality of life and self-esteem, which are superior to the control group (Q4). As mentioned above, a limited number of previous studies have yielded mixed findings regarding the effects on cognitive insight. Therefore, we do not expect changes in this domain. Trials on CR programs, as well as our own work, suggest that CR exerts a small to moderate effect on neurocognition [47, 52]. Therefore, it is expected that participants in the control group will experience greater improvements in neuropsychological functioning than those in MCT+ (Q5). Finally, we posit that participants with greater therapy motivation will have a better response (i.e., greater decrease in delusions) to MCT+ (Q6). Based on previous MCT trials [53], we hypothesize that participants with symptoms in the moderate range will benefit most from MCT+.

## Methods/Designs

### Study design

The design of the study is a multi-center, assessor-blind, parallel group, randomized controlled trial. Patients will be randomized to MCT+ or computerized CR. The study protocol has been approved by the German Psychological Society (*Deutsche Gesellschaft für Psychologie*; SM_102015_amds_div), as well as by local ethics committees (Medical Faculty of the Ruhr-University Bochum; Medical Faculty Mannheim of the University of Heidelberg and University Hospital Mannheim). It has been registered at the German Registry for Clinical Studies under the number DRKS00008001.

### Participants

The study population will consist of individuals with psychosis (as confirmed by the Mini International Neuropsychiatric Interview, 5th Ed [M.I.N.I.]) [54]. Diagnoses according to the *Diagnostic and Statistical Manual of Mental Disorders – 5th edition* (DSM-5) [55] are then derived from information gained from the M.I.N.I. To enhance recruitment and generalizability, the study is being conducted in four different German clinics for patients with psychosis: the Department of Psychiatry and Psychotherapy at the University Medical Center Hamburg-Eppendorf, the Department of Psychiatry and Psychotherapy at the Central Institute of Mental Health in Mannheim, the Clinic for Psychiatry, Psychotherapy and Preventative Medicine at the LWL University Hospital Bochum, and the Clinic for Psychiatry and Psychotherapy at the Asklepios Clinic North in Hamburg-Wandsbek. Each center has specialized out- and inpatient clinics for treatment of psychosis.

### Eligibility criteria

Generalizability of study results is of main importance; therefore, eligibility criteria were chosen to recruit a broad sample representative of the population of patients with psychosis. Participants between the ages of 18 and 65 who meet the following criteria will be included in the study: a) primary diagnosis of psychosis as verified by the M.I.N.I. and supplemented by diagnostic criteria for schizophrenia and schizoaffective disorder from the DSM-5; b) provide informed consent; c) sufficient understanding of the German language. Exclusion criteria include: a) IQ < 70 (according to a premorbid intelligence vocabulary test); b) PANSS scores ≥ 5 for hostility and non-cooperativeness (G8, P7); c) endorsement of suicidal intent; d) current alcohol or substance dependence (abstinence must be > 6 months), excluding tobacco; e) any form of documented or suspected major neurological or general medical condition that might be responsible for the psychotic manifestations; f) participation in MCT+ within the past 12 months.

### Procedure and treatment allocation

Recruitment occurs in different ways: recruitment by study staff during inpatient stays, referrals from mental health professionals at clinics both within the cooperating centers and in the community (including private practices), and by self-selection through advertisements or word-of-mouth, for example, from Internet searches for schizophrenia treatment in the community. Interested patients receive an informational leaflet and undergo a screening interview by telephone or in-person with research assistants to assess eligibility. If patients appear eligible upon initial contact, they are invited for baseline testing in which informed consent is obtained (or it is obtained before testing for patients with guardians for medical decision-making).

Blinded in-person assessments conducted by trained study research assistants, psychologists or psychiatrists are performed at baseline, 6 weeks and 6 months after baseline (see Table 1). All raters complete a rater-training workshop and also receive individual training in which they are observed as the measures are administered. The 6-week intervention phase begins directly after completion of baseline testing. To diagnose or confirm the diagnosis of psychosis or an affective disorder with psychotic features, as well as to rule-out acute suicidality and substance dependence, a semi-structured interview with the M.I.N.I. is completed with all patients at baseline and 6-month sessions. A questionnaire for intervention group preference and motivation is completed at the beginning of the first treatment session. Questionnaires assessing participants’ acceptance of both interventions are completed at post and follow-up. The sociodemographic interview and all other measures, including semi-structured interviews for schizophrenia symptoms (PANSS and PSYRATS), self-report questionnaires, and neuropsychological measures are completed at all three testing sessions (see Table 2 for list of measures and administration time points).

**Table 1:** Schedule of enrollment, interventions, and assessments

	Study period				
	Enrollment	Randomization	Intervention (12 bi-weekly sessions)	Post (6 weeks)	Follow-up (6 months)
Timepoint	T-1	T0		T1	T2
Enrollment					
Eligibility screen	X				
Informed consent		X			
Randomization		X			
Interventions					
MCT+			X		
Cognitive Remediation			X		
Assessments					
Baseline		X			
Post-intervention				X	
Follow-up					X

**Table 2:** List 1 of study measures and questionnaires

Instrument	Baseline	6 weeks (post)	6 months (follow-up)
*Diagnostic and Measures of Psychopathology*			
Sociodemographic interview	X	X	X
Mini Neuropsychiatric Interview	X		X
Positive and Negative Syndrome Scale	X	X	X
Psychotic Symptom Rating Scales	X	X	X
Calgary Depression Rating Scale	X	X	X
Personal and Social Performance Scale	X	X	X
Negative Symptom Assessment – 4	X	X	X
*Neurocognitive Measures*			
Trail Making Test A & B	X	X	X
Test d2	X	X	X
German Vocabulary Test (premorbid intelligence)	X		
Rivermead Behavioural Memory Test, Story Recall	X	X	X
*Cognitive biases*			
Fish Test	X	X	X
*Questionnaires*			
Short Form-12	X	X	X
Patient Health Questionnaire	X	X	X
Beck Cognitive Insight Scale	X	X	X
Rosenberg Self-Esteem Scale	X	X	X
Evaluation of the Intervention		X	X

A measure was developed by the authors (BS, FB, SM) to assess therapist adherence to the MCT+ manual. For patients who provide informed consent, therapy sessions are video recorded and psychologists familiar with MCT+ assess adherence. Additionally, questionnaires developed by the authors (SM) assessing participants’ acceptance of both interventions are completed after every training session attended.

Participants are allowed to use antipsychotic medications during the entire study and are not withheld from other psychosocial treatments they might be offered or are currently participating in, including participation in MCT group therapy. Participation in concurrent interventions is recorded at post- and follow-up testing sessions. Participants are allowed to withdraw from intervention or assessment sessions upon request without having to disclose a reason. All participants are compensated with 30€ upon completing of each assessment session.

### Randomization and assessor blindness

Treatment allocation is randomized via a computerized randomization plan (no stratification factors) and performed observer-blind. Extensive steps are taken to ensure rater blindness. The randomization plan is only accessible to the study coordinators, thus reducing the risk that assessors may be accidentally unblinded. Patients are informed that they are not to disclose their group assignment and are reminded of this at every testing session. At the end of the baseline testing session, all participants meet with a member of the research team who is not involved in testing and has access to the site-specific randomization list. This individual gives the participant an envelope in which intervention assignment is identified. Raters are not involved in the active intervention. Rater blinding is verified by asking the rater to guess the participant’s treatment allocation at the beginning of every testing session and indicating their certainty.

#### Outcome measures

##### Selection and diagnostic measures

The Mini-International Neuropsychiatric Interview 5.0 (German version) [54], a short, structured diagnostic interview based on DSM-5 criteria, will be conducted to assess specifically for psychosis, affective disorder with psychotic features, substance use disorders and suicidality. It is fully structured to allow administration by non-specialized interviewers. The agreement between the M.I.N.I. and the Structured Clinical Interview for DSM-IV (SCID) is good or very good for most diagnoses. Test-retest reliability is also good (kappa = .75).

##### Primary outcome measure

The PSYRATS is a commonly used semi-structured interview that measures quantitative and qualitative aspects of delusions and hallucinations, including preoccupation, conviction and distress. The difference in PSYRATS delusions subscale score from baseline to follow-up serves as the primary outcome parameter. The validity of the PSYRATS has been demonstrated in chronically psychotic patients and it has been shown to have good psychometric properties [56]. Unlike the PANSS, which collapses important aspects of positive symptoms (e.g., delusion conviction and distress), the PSYRATS allows for a multivariate assessment of delusions and, the PSYRATS is highly correlated with the PANSS.

##### Secondary outcome measure

1. The Positive and Negative Symptoms Scale (PANSS) is a 30-item scale that assesses positive, negative and general psychopathology based on a semi-structured interview. The PANSS has good psychometric properties and is sensitive to change [57].
2. The Fish Task [58] is a computerized variant of the classical Beads Task. In this measure, participants are shown two lakes containing orange and gray fish (e.g., lake A with 80 % orange and 20 % gray fish, and lake B with the reverse ratio). Ten fish are successively presented in a predetermined sequence to the participant. After each draw, the participant indicates whether they can make a decision regarding the origin of the fish. All fish drawn remain visible throughout the task in order to minimize working memory demands. Parallel versions are used across the testing sessions to reduce practice effects. The presence of a jumping to conclusions bias is defined as reaching a decision after drawing only one or two fish. The key variable of interest is the number of draws to decision.
3. The Negative Symptom Assessment (NSA-4) [59] is an instrument to rapidly assess negative symptoms based on a rating by the interviewer, which includes four items (restricted speech quantity, reduced emotion, reduced social drive, and reduced interests). Each item is rated by the interviewer on a scale from ‘1’ to ‘6’ where ‘1’ represents no reduction from normal behavior and ‘6’ represents severe reduction in or absence of the behavior, with markedly impaired functionality. Consistency of ratings by experts has been shown to be high [60].
4. The Patient Health Questionnaire-9 item (PHQ-9) [61] is a self-report measure of depression, which is a module of the full Patient Health Questionnaire. Each item is rated based on the presence or absence of the symptom over the past 2 weeks for a total possible score of 27. Mild depression is likely at a score of 5, while the cutoff for severe depression is 15. Internal reliability (Cronbach’s α = .89) and test-retest reliability (*r* = .84) of the scale are excellent.
5. The Calgary Depression Scale for Schizophrenia (CDSS) [62] is a brief semi-structured interview scale, which allows raters to assess depressive symptoms separate from positive, negative and extrapyramidal symptoms in people with schizophrenia. The nine items of the scale are rated on operational criteria from ‘0’ to ‘3’. The scale is correlated with other depression rating scales (Hamilton Depression Rating Scale and the Beck Depression Inventory). Internal reliability is good (Cronbach’s α = .79) [63].
6. The World Health Organization Quality of Life - BREF (WHOQOL-BREF) [64, 65] is a self-report measure of overall life satisfaction. The measure assesses quality of life in four domains: physical, psychological, social and environment. Internal consistency assessed internationally across several study centers for most scales is good (Cronbach’s α > .70).
7. The Rosenberg Self-Esteem Scale (RSE) [66] is a widely used 10-item self-report measure that assesses global self-esteem. Internal consistency (Cronbach’s α = .84) as well as split-half reliability (*r* = .74) of the German version are good.
8. The Beck Cognitive Insight Scale (BCIS) [67] measures self-reported ability to distance oneself from and reflect upon subjective cognitive biases. The 15-item measure is comprised of two subscales: self-reflectiveness and self-certainty. Insight plays an important role in the formation and maintenance of psychotic symptoms. Internal consistency among individuals with schizophrenia is acceptable (Cronbach’s α = .60 – .68). In Beck’s initial study, the mean self-reflectiveness score for patients without a psychosis diagnosis did not significantly differ from those without a psychosis diagnosis while self-certainty was higher among those with a psychosis diagnosis in comparison to patients without a psychosis diagnosis [67].
9. The Personal and Social Performance Scale (PSP) [68] is a semi-structured clinician-rated interview of social functioning over the past 30 days in individuals with schizophrenia. The PSP rates functioning on four subscales: socially useful activities, personal and social relationships, self-care, disturbing and aggressive behavior. Descriptive anchor points (I = absent; VI = very severe) are used to rate patient functioning in these domains. An overall functioning score is then derived from the subscale scores. Inter-rater reliability is acceptable (Cohen’s κ = .68).
10. The Short Form-12 (SF-12) [69] is a short self-report questionnaire used to measure functional health and well-being. The SF-12 has been shown to distinguish individuals with severe mental illness from the general population. Psychometric properties of the scale, including test-retest reliability and convergent and divergent validity among individuals with severe mental illness are supported [70].

Additionally, to characterize the sample and assess change in neuropsychological functioning, neuropsychological assessment measures will be administered, including the Trail Making Tests A and B (mental flexibility, set-shifting) [71], Test d2 (selective attention) [72], Story Recall subtest of the Rivermead Behavioural Memory Test (short- and long-term verbal memory) [73], and the German Vocabulary Test (*Wortschatztest*; premorbid intelligence) [74].

#### Intervention

To ensure comparability of results, both interventions are implemented for the same duration (6 weeks; 12 sessions) and frequency (45–60 min per session). Psychologists undergoing post-graduate training provide both MCT+ and computerized CR. All MCT+ trainers completed a 2-day MCT+ workshop and receive regular intervision from two of the authors of the MCT+ therapy program (RV, FB).

##### Individualized metacognitive therapy program (MCT+)

The main aim of MCT+ is to “plant the seed of doubt” regarding rigid delusional convictions and to encourage patients to critically reflect, extend and change their approach to problem solving. Importantly, patients are not to be “talked out” out their symptoms as this can lead to an escalation of cognitive biases and, thus, increased symptoms. Rather, symptoms are first normalized and an individualized plan for addressing symptoms and cognitive biases is formulated based specifically on each patient’s presenting problems and goals. The second edition of the German manual by Moritz et al. was provided to all study trainers (MCT+ materials can be obtained by contacting the authors) [75]. The major changes to the updated manual are the addition of elements regarding stigma and of a new unit focused on self-esteem. An overview of the MCT+ units is provided in Table 3. All of the MCT+ therapy units that focus on cognitive biases (i.e., Therapy Unit 4: Attributional Style; Therapy Unit 5: Decision Making; Therapy Unit 6: Changing Beliefs; Therapy Unit 7: Empathizing; Therapy Unit 8: Memory and Certainty of Judgment) first highlight the fallibility of cognition in general and discuss specific problematic exaggerations of these cognitive processes in individuals with schizophrenia. In a second step, the relationship between cognitive biases, symptoms and their consequences for everyday life, as well as the course of the illness, are emphasized.

**Table 3:** MCT+ therapy units

Unit	Description	Exercise/Therapy worksheet examples
1. Case History Questionnaire	The patient’s case history including current problems and symptoms, as well as previous treatments, are gathered through a clinical interview. The patient’s level of motivation is evaluated and the importance of the therapeutic relationship is discussed.	With the use of worksheets, the therapist and patient identify the patient’s current problems, including delusions or hallucinations and to what extent the patient is certain of this belief. Interpersonal or coping difficulties resulting from the delusion/hallucination are identified.
2. Introduction to MCT+	Information about MCT+ and the main therapeutic strategies are discussed. A secondary goal is to develop an understanding of the patient’s problem areas and possible therapeutic targets.	An overview of the therapy program is provided and the patient’s goals are determined with help from therapy worksheets. Patients are asked to identify current or past symptoms and how distressed they are (have been) by these symptoms.
3. Case Formulation	An individual vulnerability-stress-model is created utilizing a “fire” metaphor. For patients with limited illness insight who may be overwhelmed by such a discussion or may be unable to accept explanations for their symptoms, this therapy unit can also be completed toward the end of the training.	Using therapy worksheets the “fire” metaphor is personalized. Vulnerability factors (e.g., genetic predisposition) are compared to wood or coals, while factors that contribute to a first psychotic episode (e.g., stress), are likened to a spark, which ignites the flames. Interventions, which help to reduce symptoms (e.g., psychotherapy), are the “fire extinguisher.”
4. Attributional Style	The goal is to help patients understand that complex social situations can rarely be attributed to one cause (i.e., mono-causal attributions), but are rather the outcome of many factors (myself, other people, circumstances). It is important to help patients gain insight to situations in which they themselves tend to make one-sided attributions, and how certain attributions (especially personalization and blame) reinforce delusions.	Using fictional and, if possible, personal real-life examples, patients are encouraged to identify multiple causes of events that may otherwise be attributed to psychosis-related factors (e.g., a strange smell means that I am being poisoned rather than the possibility that there was a chemical spill or I am in an unfamiliar area where smells may be new). A “card trick” exercise is used to illustrate that there are often simple explanations for strange experiences.
5. Decision Making	Jumping to conclusions can lead to suboptimal decisions, sometimes with dramatic consequences. Patients are encouraged to reflect on whether they currently, or in the past, have made decisions too hastily, and if their delusions were caused or strengthened by these “short-circuit” thinking patterns. Patients should learn to consider the pros and cons of making strong assumptions and to always be open to alternative explanations.	Patients are shown, for example, slides of line drawings in which the details of the figure become increasing clear with each slide. The patient is asked to indicate when they are certain that they know what is depicted. Early responses are often incorrect as not enough details are present to reach a clear decision.
6. Changing Beliefs	Being stubborn and inflexible at times is part of human nature and can actually be helpful to ensure a certain amount of stability so that we do not instantly question everything and everyone. However, being overly fixated can lead to problems too, especially if one’s convictions are wrong.	Pictures, which depict a story, are shown to the patient in reverse order (i.e., the last pictures is shown first). The patient is asked to indicate when they are certain that they know the correct beginning of the story. Often reaching a decision with incomplete information leads to an incorrect response.
7. Empathizing	“To err is human”, especially when it comes to assessing the motives of others. It is emphasized that we cannot infer what another person’s emotional state is from facial expressions or nonverbal signals alone. To become more certain about our interpretation, patients are encouraged to consult additional sources (e.g., contextual factors, previous knowledge of the person). Patients learn that social perceptions are often influenced by personal feelings and people often tend to confuse their inner emotional world with the outside world (e.g., when anxious, a patient may think: “Everybody is against me”). "Unwritten social rules” are also discussed with the patient. The relationship to delusions should be carefully clarified.	The patient is shown a series of pictures and is asked to guess how the person is feeling or what they are thinking. It is emphasized that it is often difficult to know how another person is feeling and that additional information should be sought to avoid making incorrect assumptions. The patient is asked to identify situations in which they were uncertain of how others were feeling.
8. Memory and Certainty of Judgment	The potential for memory biases are discussed and normalized. It is always important to consider the possibility that vague memories might be false, and therefore, should be investigated further by asking others or consulting documents as false memories can have potentially serious consequences (e.g., conflict).	Patients are briefly shown complex scenes with many objects and are then asked to identify which objects were present in the scene. It is discussed with the patient that, like all humans, their memory may not always be perfect and details are often added or omitted based on previous experiences or “common sense” (i.e., false memories).
9. Depression and Thinking	This unit focuses on improving self-perceptions and improving or maintaining social relationships with others. Depression is not “unavoidable” or innate, but tends to be promoted and maintained through certain thought distortions, which can be changed. Therapists help patients to become more aware of if and when they tend to have depressive thought distortions and how their personal depressive symptoms or delusions were intensified or perhaps caused by such thoughts. The extent to which psychosis has an effect on self-esteem is also discussed.	With the help of fictional and, if possible, personal real-life examples, patients are asked to identify alternative, more helpful thoughts for various situations when one might use “black and white thinking” or “should” statements (e.g., when receiving negative feedback from a boss).
10. Self-Esteem	Self-esteem is defined in MCT+ as something that one largely subjectively determines and actually has little to do with the opinions of others. The therapist carefully addresses the different influences that psychotic symptoms can have on one’s self-esteem (excitement and a feeling of being important versus anxiety and guilt), while also recognizing that these symptoms can also partially serve a social function. Using the metaphor of an “inner critic” and a “well-meaning companion,” it is carefully suggested that sometimes content from auditory hallucinations can reflect inner conflicts.	The patient is encouraged to identify personal strengths, especially in areas, which are often not noticed or thought of as self-evident. Concrete situations in which these strengths have been demonstrated are identified.
11. Dealing with the Diagnosis and Relapse Prevention	Based on information gained throughout the 6 weeks, patients are given information about their disorder and how to cope with the diagnosis in everyday life, especially regarding communication of information about the disorder in social situations. Patients are made aware of possible ways the disorder may progress. It can also be beneficial to involve relatives in this final session because they often recognize prodromal symptoms earlier on than patients.	An emergency plan is created in cooperation with the patient, which clarifies whom the patient should seek in case of a crisis (e.g., an institution that the patient trusts, a therapist). Stress reducing and coping strategies are discussed. It is also discussed with the patient in which situations revealing their diagnosis may be helpful or unhelpful.

Although the MCT+ units are presented in the order provided in Table 3, it is emphasized that treatment should be tailored to the individual needs and insight level of the patient, as well as trainer preferences. As such, more time can be spent on any one unit and themes can be skipped completely. For the purposes of the RCT, all trainers were instructed to complete the first therapy units focused on discussion of the importance of trust and openness to the therapeutic relationship and taking a case history (Therapy Unit 1), introducing MCT+ (Therapy Unit 2) and developing a personal illness model (Therapy Unit 3; when appropriate, this unit can also be completed at a later stage during therapy). In addition, trainers are to choose at least two units addressing cognitive biases. There are also two units devoted to affective problems (Therapy Unit 9: Depression and Thinking; Therapy Unit 10: Self-Esteem). In the last unit, focus is on coping with (the stigma of) illness, preparing patients for the discontinuation of therapy and discussing relapse prevention strategies (Therapy Unit 11). Each unit includes a number of therapy demonstrations, as well as worksheets displaying discussion topics and exercises that serve to illustrate the main topic. Certain exercises (e.g., card trick in Therapy Unit 4) are preferably presented with the help of a computer laptop or tablet.

In addition, most sessions include “between-session” tasks (i.e., homework) to encourage application and practice of skills in daily life. Homework is then discussed at the beginning of the following session. Given that many patients with psychosis have difficulties remembering and organizing information, in the last session participants are given a booklet of copies of important MCT+ therapy demonstration sheets that were presented during the treatment so that they have a “self-help” booklet to review after therapy has ended. Additionally, postcards are sent to the participants three months after the final session to inquire whether they have been using the skills learned in MCT+. Although participants are not allowed to respond to the mailing, they are encouraged to look at their booklets and review the MCT+ materials.

##### Cognitive remediation

MyBrainTraining^©^ (BBG Entertainment GmbH) [76] is a newly developed computer-based cognitive remediation program, which aims to improve cognitive functioning deficits present in schizophrenia by presenting a series of cognitive exercises, which are repeatedly practiced. In the present study, participants complete all exercises independently. The program consists of thirty exercises aimed at training executive functioning through different types of tasks, including those of vision, arithmetic, logic and memory. All exercises are conducted on PC’s over the Internet. The exercises were designed during the development of the “Train your Brain with Dr. Kawashima” program in cooperation with the Industry University Research Project and Professor Dr. Ryuta Kawashima.

Based on the participant’s performances, the difficulty of each task automatically increases or decreases. The exercises are similar to video games and include fun or motivational elements. The administrator has the ability to determine specific training plans and to adapt exercises to each patient’s needs (e.g., level of difficulty, varied time limits, etc.). Data protection and security comply with industry standards.

For the present study, participants are asked to complete the exercises in a specific order and are provided with feedback from the program on their performance, which they record on an exercise log. Log-in information is not shared with participants to prevent practice at home. Like the MCT+ group, three months after the final session participants are sent a postcard asking if they are still using the skills learned while completing MyBrainTraining^©^ and if they have continued cognitive exercises using other modalities (e.g., crossword puzzles, Sudoku, etc.).

#### Sample size

**Fig. 1:**
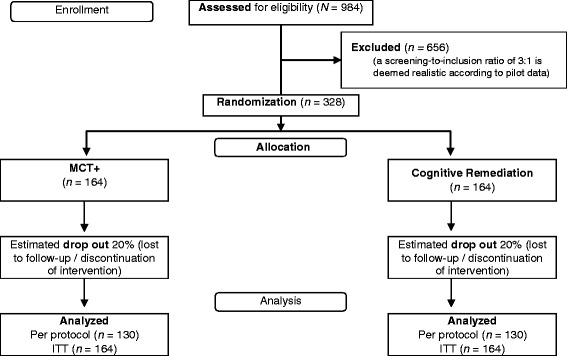
Study flow

This is the first trial of MCT+ in which participants are followed for a longer period after completion of treatment. As such, determination of optimal sample size was based on an initial trial of MCT+ [34], which included 48 patients and yielded medium to large effect sizes (ɳ_partial_^2^ = .08 – .13) compared to cognitive remediation (CogPack^©^). In this pilot study, non-compliance rates were low over the 4-week follow-up (4 %) and patients’ acceptance was excellent. Most patients eligible for treatment agreed to participate in the study, although 26 % refused participation. Non-completion rate from a 6-month follow-up of group MCT was 14 % after six months, although the greatest number of participants (10 %) dropped out during the first four weeks of the study (from baseline to the post-treatment follow-up). Based on this previous work, we anticipate a completion rate of 80 % for the primary endpoint (T0-T2). See Fig. 1 for a flow chart of the recruitment and study procedure.

Using calculation software PASS 2008 [77] with a conventional alpha level of .05 and a beta-value of .20 for a two-tailed test, it was calculated that each group must be comprised of 130 patients to detect a small to medium effect (*d* = 0.35) at the 6-month follow-up testing. Assuming a dropout rate of 20 % (including dropout due to non-compliance and loss to follow-up), the study will be conducted with a sample size of 328 patients or 82 patients per trial center.

#### Statistical analyses

Two sets of statistical analyses will be performed. First, on an intention-to-treat (ITT) basis, which considers all patients with available baseline data. An expectation maximization (EM) algorithm will be used to estimate missing values. The second set of analyses will include only participants with baseline and post and/or follow-up data (per protocol, PP). An ANCOVA model will be conducted with group (MCT+/MyBrainTraining^©^) difference between T0 and T1 as the dependent variable. Both treatment (independent of time) and condition*time interaction effects will be evaluated. Change scores between T0 and T2 for the primary outcome, as well as analyses over both time points (T0 vs. T1 and T0 vs. T2) for secondary endpoints (PANSS syndrome scores, Fish Task, PHQ-9, CDSS, NSA, WHOQOL-BREF, RSE, BCIS, PSP, neuropsychological outcomes), will be tested using analogous models. Baseline performance or symptoms, antipsychotic drugs (% of maximum dosage) and concurrent treatment (e.g., group MCT, individual psychotherapy, and occupational therapy) will be included in the models as covariates to explore possible impacts on treatment outcome. Secondary analyses will be conducted to examine change between T1 vs. T2 for primary and secondary outcomes.

A logistic regression will be used to analyze variables that may identify participant response to MCT+, including severity of baseline psychotic symptoms, level of motivation for treatment, satisfaction with group assignment, baseline depression, and number of sessions completed. Two groups will be defined (criterion: decline of 25 % or more points on the PSYRATS from T0 to T2) and non-responders. No interim analyses will be conducted. In a sensitivity analysis, we will explore the effect of missing values using various imputation methods (best and worst observation carried forward, last observation carried forward, full-informational maximum likelihood).

## Discussion

This is the first large-scale multi-center trial investigating the immediate and mid-term effects of the individualized metacognitive therapy program for psychosis compared to an active control group. Although small to moderate effects have been found for other psychotherapeutic interventions, particularly CBTp, implementation rates of empirically-supported therapies remain low and a large treatment gap remains for patients with schizophrenia [14, 15, 18]. MCT+ represents an alternative low-threshold approach, which addresses (mainly) positive symptoms, but more recently also depression and self-esteem, which may be able to reach patients who otherwise may “fall through the cracks” of the healthcare system [14, 15, 18]. MCT+ may also serve as an add-on approach for patients who are currently participating in therapy or are only receiving medication.

Retention and recruitment of patients is a major challenge for all studies involving individuals with schizophrenia. Many individuals with schizophrenia tend to feel stigmatized [78, 79], have poor insight [80], be distrustful of care providers [81] and have cognitive limitations, including deficits in memory and executive functioning [82] that lead to problems remembering appointments. Therefore, designing a study, in which these barriers are minimized and patients enjoy participating is essential. As previously mentioned, MCT and MCT+ are well-accepted by patients [30, 34, 35]; on average, patients attend 80 % of the MCT+ sessions [34]. In our most recent study on group MCT, completion rates at 6 months and 3 years were 86 % and 62 % respectively [47]. To maximize completion among patients in our control group, we carefully chose an active intervention (computerized CR), which is designed much like a computer game. Similar programs have been well-accepted by patients in previous studies [34, 83]. Nonetheless, to improve completion rates and enhance memory for material in the present study, at the end of the 6-week training, we provide patients in the MCT+ group with a packet of therapy demonstration sheets that were discussed in therapy sessions. Additionally, patients in both groups are mailed a postcard three months after completion of the post assessment to remind them of the strategies they learned during the study. Patients are also reminded both via telephone and mail of upcoming testing sessions. Many patients are recruited directly during an inpatient stay and through outpatient providers, such as therapists and psychiatrists at the patient's treatment center, which helps to decrease feelings of distrust since research staff are part of the patient’s current healthcare facility.

Strengths of this RCT are the large sample size and non-stringent participation criteria, which aim to improve generalizability of results (e.g., inclusion of participants who may meet criteria for alcohol or substance abuse). In light of research indicating that patient preferences are often not considered in psychotherapeutic settings and that mismatch between therapist and patient priorities contributes to attrition rates (Moritz S, Berna F, Jaeger S, Westermann S, Nagel M. The customer is always right? Subjective target symptoms and treatment preferences in patients with psychosis, submitted), we emphasize the importance of choosing MCT+ units that match the patient’s needs. In this way MCT+ is not a “one size fits all” approach, but rather offers a personalized treatment plan while maintaining its low-threshold structure. Additionally, as most trials on MCT and MCT+ have been conducted at the University Medical Center Hamburg-Eppendorf, inclusion of other study centers will serve to reduce potential allegiance effects. Given the “add-on” nature of MCT+, participants are also allowed to continue routine antipsychotic treatment including medication and even group MCT. However, we carefully monitor treatments that patients are concurrently participating in, and ask at the beginning of the study which interventions they have previously participated in. We also do not influence medication, although information on substances and dosages is gathered at each testing visit.

The use of MyBrainTraining^©^ as a control condition prevents the danger of similar interventions in both conditions, which would be the case if CBTp had been chosen. Similar to other therapy studies, the effect of therapeutic relationship cannot be completely controlled. Additionally, since all patients are allowed to continue TAU and many are recruited directly from an inpatient treatment program, effects of MCT+ may be partially masked for individuals already receiving intensive comprehensive care.

## Conclusions

If MCT+ is shown to be superior to MyBrainTraining^©^ in reduction of positive symptoms of psychosis, particularly delusional ideation, MCT+ may serve as a valuable low-threshold therapy for patients and a first step towards comprehensive treatment. Additionally, dissemination is facilitated as materials in most languages are available as free downloads via the MCT+ website [75]. As such, this intervention could overcome traditional treatment barriers to help close the treatment gap for individuals with psychosis and provide clinicians with an alternative to therapies, which may be too costly or time-intensive to routinely implement in daily clinical practice.

## Trial status

The first participant was enrolled in July 2015. At the time of submission of this study protocol participants were still being recruited.

## Abbreviations

CBTp: cognitive behavioral therapy for psychosis
CR: cognitive remediation
EBTs: evidence based treatments
MCT: metacognitive training (group)
MCT+: individualized metacognitive therapy program
RCT: randomized controlled trial
TAU: treatment-as-usual
